# Digging for Stress-Responsive Cell Wall Proteins for Developing Stress-Resistant Maize

**DOI:** 10.3389/fpls.2020.576385

**Published:** 2020-09-25

**Authors:** Liangjie Niu, Lunyu Liu, Wei Wang

**Affiliations:** College of Life Sciences, National Key Laboratory of Wheat and Maize Crop Science, Henan Agricultural University, Zhengzhou, China

**Keywords:** cell wall, stress-responsive proteins, abiotic and biotic stresses, stress-resistant crops, *Zea mays*

## Abstract

As a vital component of plant cell walls, proteins play important roles in stress response by modifying the structure of cell walls and involving in the wall integrity signaling pathway. Recently, we have critically reviewed the predictors, databases, and cross-referencing of the subcellular locations of possible cell wall proteins (CWPs) in plants (*Briefings in Bioinformatics* 2018;19:1130–1140). Here, we briefly introduce strategies for isolating CWPs during proteomic analysis. Taking maize (*Zea mays*) as an example, we retrieved 1873 probable maize CWPs recorded in the UniProt KnowledgeBase (UniProtKB). After curation, 863 maize CWPs were identified and classified into 59 kinds of protein families. By referring to gene ontology (GO) annotations and gene differential expression in the Expression Atlas, we have highlighted the potential of CWPs acting in the front line of defense against biotic and abiotic stresses. Moreover, the analysis results of cis-acting elements revealed the responsiveness of the genes encoding CWPs toward phytohormones and various stresses. We suggest that the stress-responsive CWPs could be promising candidates for applications in developing varieties of stress-resistant maize.

## Introduction

Crops, such as maize, wheat, and rice, are cultivated Worldwide as staple food. Under field conditions, crops are subjected to various abiotic stresses (e.g., drought, cold, salt, and heat) and biotic stresses (e.g., pests and pathogens) (Baillo et al., 2019), which affect plant growth and crop yields. Since the demand for food increases as the population grows, developing stress-resistant crops is recognized as the most efficient way to improve crop yields under stress conditions (Zhang H. et al., 2018; Eshed and Lippman, 2019).

As a supplement to conventional breeding programs, molecular and transgenic technology is a promising strategy for developing stress-resistant crops, particularly by engineering multiple transgenes for the introduction of whole metabolic pathways (Bock, 2013). Thus, it is vital to discover stress-related genes that can be used for improving the resistance of crops. The resource for the known stress-related genes is still limited (Zhang H. et al., 2018). For example, in maize, only a few resistant genes have been identified and modulated to improve resistance and yield (Nelson et al., 2007; Virlouvet et al., 2011; Habben et al., 2014; Nuccio et al., 2015; Zuo et al., 2015; Wang et al., 2016).

The cell wall is an outermost layer of plant cells exposed to different environments, and it provides structure, support, and protection to plants (Houston et al., 2016; Kesten et al., 2017). Its role is substantially affected by the activity of cell wall proteins (CWPs) that account for 10% of the dry mass of primary cell walls (Wolf et al., 2012; Lin et al., 2017). It is usually considered that CWPs act in the front line of apoplastic defense mechanisms (Cassab and Varner, 1988). They can modify the structure of cell walls and are involved in the wall integrity signaling and innate immunity under stress conditions (Zagorchev et al., 2014; Gust et al., 2017; Tucker et al., 2018; Vaahtera et al., 2019). However, the potential of CWPs for improving the resistance of crops has not yet been discussed on a proteome-wide scale.

In this study (released partly as a pre-print at bioRxiv, Niu and Wang, 2020), taking the model crop plant maize as an example, we dig for stress-responsive maize CWPs in the UniProt KnowledgeBase (UniProtKB). We have highlighted the potential of CWPs acting in the front line of defense against biotic and abiotic stresses. The knowledge thus learned may facilitate the research of identifying novel stress genes/proteins for developing stress-resistant maize.

## Discovering Stress-Responsive CWPs by Proteomic Analysis

Most of CWPs have basic p*I* values, with a signal peptide, and are modified post-translationally, particularly *via* hydroxylation and glycosylation (Albenne et al., 2013). After the recognition of the signal peptide, CWPs synthesized in cytosol are secreted from endoplasmic reticulum, Golgi apparatus, and plasma membrane to the cell wall and/or extracellular space (Wu et al., 2018).

CWPs are low abundance proteins in the whole cell proteome (Jamet et al., 2008). Two different approaches, namely nondestructive and destructive, are used to isolate loosely bound CWPs (including those present in the intercellular space) and tightly bound CWPs, respectively (Lee et al., 2004; Feiz et al., 2006; Jamet et al., 2008; Albenne et al., 2013). By performing the nondestructive method based on the vacuum infiltration-centrifugation technique, water-soluble and loosely bound CWPs were extracted from maize roots (Zhu et al., 2006; Zhu et al., 2007). Moreover, different infiltration solutions extracted different subsets of apoplast proteins from maize leaves (Witzel et al., 2011). By the destructive method, cell walls were partially purified from ground plant materials, and sequentially extracted in buffers with different ionic strengths (Printz et al., 2015; Canut et al., 2017). As such, the obtained CWP profiles from different methods were complementary (Sergeant et al., 2019).

The proteome-wide differential analysis of two tolerant-contrast varieties, particularly isobaric tags for relative and absolute quantitation (iTRAQ)-based approaches can provide quantitative variations in protein abundance under stress conditions (Zhu et al., 2007; Zhang H. et al., 2018; Jia et al., 2020; Kruse et al., 2020) and are widely used to discover stress-responsive proteins for the improvement of crop resistance. In these studies, only a fewer number of stress-responsive CWPs have been identified (Bhushan et al., 2007; Pandey et al., 2010; Hu et al., 2015). This is mainly due to the following facts: the isolation of wall fractions is often contaminated by intracellular proteins (Jamet et al., 2008); the low abundance of CWPs (especially in the extracellular space) may escape extraction and identification (Zagorchev et al., 2014); and the post-translational modifications and stress-induced production of reactive oxygen species (ROS) greatly affect the association of the extracellular CWPs to the wall. Therefore, it is difficult to isolate and discover stress-responsive CWPs *via* conventional proteomic approaches.

Over the last decade, genome sequencing (Schnable et al., 2009; Jiao et al., 2017) and high-throughput profiling analysis (Chivasa et al., 2005; Zhu et al., 2007; Zheng et al., 2009) in maize have generated huge CWP data, which have been stored in UniProtKB. Therefore, exploring stress-responsive CWPs from the sequences stored in UniProtKB will provide a lot of gene resources that could be used in improving the stress tolerance of crops.

## Digging for the Stress-Responsive CWPs in Maize Recorded in UniProtKB

UniProtKB is the central hub for the collection of functional information on proteins, in which the proteins have either been confirmed *via* experimental evidence or entirely predicted (Schneider et al., 2012). GO of CWPs involves cell wall (GO:0005618) proteins and apoplast (GO:0048046) or secreted proteins. The apoplast is the extracellular space outside the plasma membrane consisting of cell wall and intercellular space. Thus, the apoplast proteins stand for the generalized CWPs. Using the keywords “*Zea mays*+ cell wall or apoplast or secreted protein”, we retrieved 1,873 possible maize CWPs, with only 50 curated entries, on UniProtKB (March 5, 2020). Many sequences were redundant in UniProtKB because different maize lines have been sequenced and submitted separately (Schnable et al., 2009; Jiao et al., 2017). After deleting redundant and incomplete sequences, the remaining sequences were evaluated as per the subcellular locations. Those proteins without location annotation were predicted on the online server HybridGO-Loc (http://bioinfo.eie.polyu.edu.hk/HybridGoServer/; Wan et al., 2014), as previously recommended (Wu et al., 2018). Finally, only those entries with cell wall or extracellular locations were kept for further analysis.

**Figure 1:**
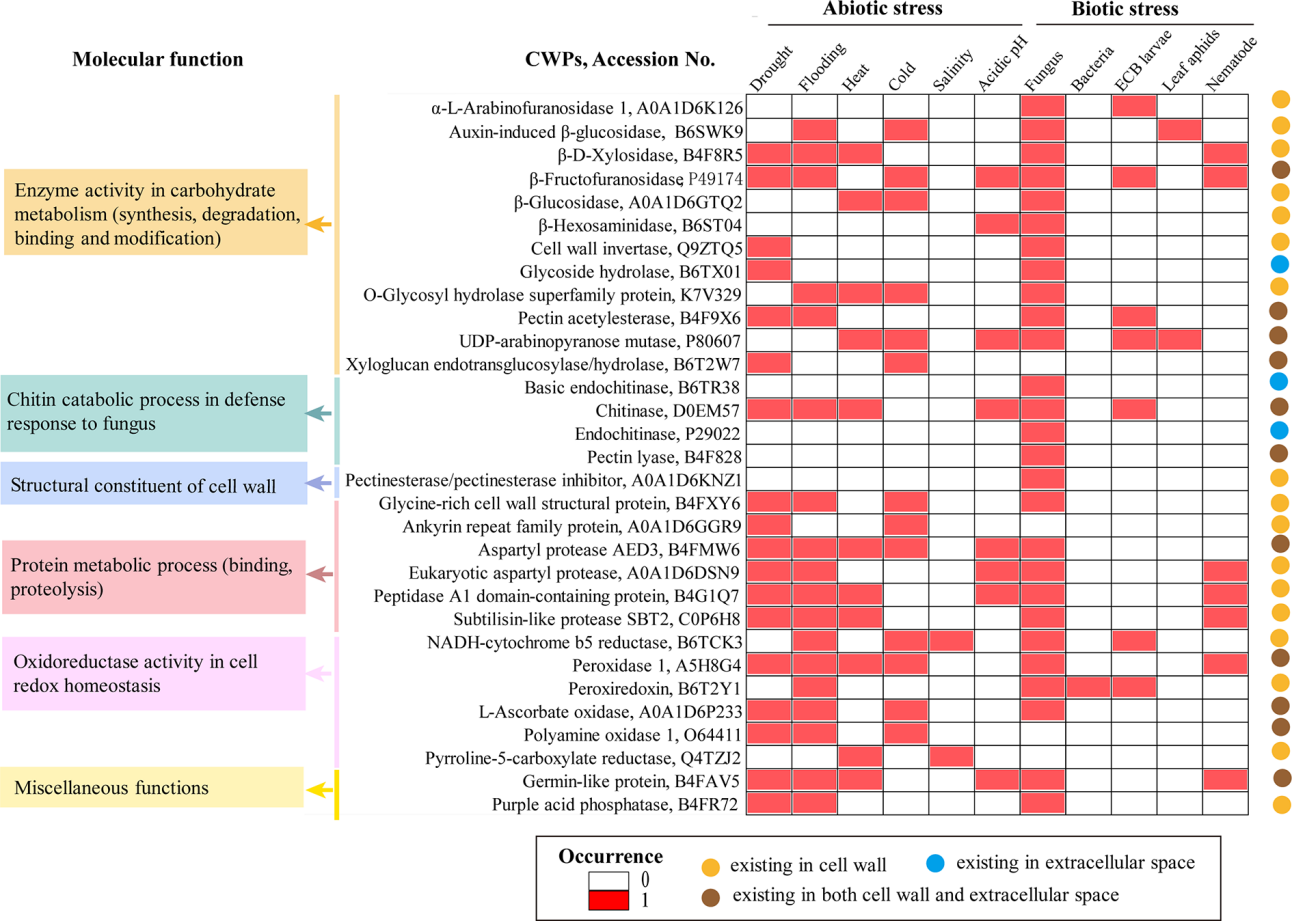
Molecular function, subcellular location, and possible roles of representative Maize cell wall proteins (CWPs) in stress responses. The subcellular location of proteins is asserted according to the annotation in UniProtKB or predicted using the server HybridGO-Loc. The molecular function and involvement of CWPs in a specific stress were summarized according to the gene ontology (GO) annotation and gene differential expression in Expression Atlas

The maize CWP dataset includes 863 protein sequences, belonging to 56 kinds of protein families; 70.5% of the protein sequences (608/863) are annotated with cell wall or extracellular locations in UniProtKB (**Supplementary dataset 1**). The top 10 families are expansin (109), pectinesterase (108), xyloglucan endotransglucosylase/hydrolase (80), peroxidase (82), polygalacturonase (69), pectin acetylesterase (66), α-L-arabinofuranosidase (64), pectin lyase (51), germin-like proteins (42), and galactosidase (27). Clearly, as found in root tips, the proteome composition of the maize cell walls shows high diversity with spatial variations (Zhu et al., 2007). Regarding the subcellular locations, 32 kinds of CWPs are found only in the cell wall, 11 kinds are found only in the extracellular space, and 13 kinds are found both in the cell wall and the extracellular space (**Figure 1**). Numerous CWPs, such as peroxidase, malate dehydrogenase, purple acid phosphatase, NADH-cytochrome b5 reductase, and peroxiredoxin, also have intracellular locations.

Different from the type I cell walls in Arabidopsis, maize and rice share the type II cell wall of grasses, i.e., the cell walls have a framework of cellulose microﬁbrils cross-linked primarily with glucurono-arabinoxylans (Penning et al., 2009). Therefore, we compared the cell wall proteome composition of maize and rice (**Table S1**). The rice CWPs were searched in WallProtDB (http://polebio.lrsv.ups-tlse.fr/WallProtDB/), which is a specialized collection of cell wall proteomic data (San Clemente and Jamet, 2015) with 2,170 protein sequences from 11 different plant species (except maize). As a result, 270 rice CWPs were retrieved, belonging to at least 31 kinds of protein families. Thus, the number of rice CWPs was less than that of maize CWPs because rice CWPs were obtained from the proteomic analysis.

The detailed comparison revealed similar functional categories between maize and rice CWPs: both include proteins acting on cell wall polysaccharides, oxidoreductases, proteases, structural proteins, signaling proteins, and miscellaneous proteins, but differed in the kinds of CWPs (**Table S1**). Maize had numerous CWPs (e.g., α-L-arabinofuranosidase, exopolygalacturonase, galactosidase, pectin acetylesterase, pectin lyase, and polygalacturonase) involved in the formation and modification of the wall and in the defense response toward fungus (e.g., chitinases). Particularly, α-L-arabinofuranosidase is abundant in the type II cell walls of maize (Zhu et al., 2006), but it is not recorded in rice CWPs. Similarly, several rice CWPs had no homologous proteins in maize, such as thaumatin, and those related to lipid metabolism. This is possibly due to the differences in physiology and biochemistry of both species: maize is a typical C4 grass, whereas rice is a typical C3 grass. Moreover, corresponding CWPs of maize and rice shared high identities, such as aspartyl protease (B4FMW6 vs. Q6F4N5, 89.4%), expansin (A0A1D6HK98 vs. Os02g0744200, 83.9%), germin (A0A1D6L886 vs. Os03g0804500, 71.4%), glycoside hydrolase (B6TX01 vs. Os03g0124900, 84.7%), peroxidase (A5H8G4 vs. Os01g0326000, 71.7%), and purple acid phosphatase (B4FR72 vs. Os12g0637100, 54.15%).

## The Potential Roles of Stress-Responsive CWPs

To explore the potential roles of maize CWPs in stress responses, we examined the gene differential expression of maize CWPs in Expression Atlas (https://www.ebi.ac.uk/gxa/), which reports the experimentally proven effects of inducers and repressors on the level of mRNA expression. As a result, 36 kinds of maize CWPs are found to respond to various stresses, with a distinct set of CWPs responding to biotic and abiotic stresses (**Table S2**). Particularly, 17 kinds of CWPs, such as chitinase, eukaryotic aspartyl protease, β-fructofuranosidase, germin-like protein, O-glycosyl hydrolase superfamily protein, NADH-cytochrome b5 reductase, peptidase A1 domain-containing protein, peroxidase 1 and subtilisin-like protease SBT2.6, UDP-arabinopyranose mutase, and β-D-xylosidase, are potentially involved in multiple stresses (**Figure 1**).

Stress-responsive CWPs may have direct roles in stress resistance, such as anti-pathogen (chitinases), ROS scavenging (peroxidases), glycoside hydrolase family proteins, and oxidoreductases. Glycoside hydrolase family proteins (e.g., chitinase) hydrolyze chitin (a primary component of a fungus cell wall) to confer crops resistant toward fungi (Wang et al., 2019). Oxidoreductases (e.g., peroxidase, L-ascorbate oxidase, malate dehydrogenase, and polyamine oxidase) can maintain cell redox homeostasis that may change under stress conditions (Passardi et al., 2004; Zhu et al., 2007).

CWPs may also have indirect roles in enhancing the cell wall structure, such as cell wall structural proteins and the enzymes involved in the organization and modification of the cell wall. The importance of these CWPs is obvious because polysaccharides are the largest components of plant cell walls and are constantly subjected to remodeling during plant development or during response toward environmental cues (Tenhaken, 2014; Houston et al., 2016).

CWPs can be stress messengers; for example, leucine-rich repeat family proteins that can perceive and transduce extracellular stress signals. The STRING analysis suggested that maize CWPs participate in mitogen-activated protein kinase (MAPK) and wingless-related integration site (Wnt) signaling pathways. It is recognized that MAPK and Wnt signaling pathways may play pivotal roles in the linking perception of external stimuli with changes in the cellular organization or gene expression (Taj et al., 2010). In addition, numerous protein/protein interactions are expected among CWPs, between CWPs, and those spanned in the plasma membrane. The STRING analysis revealed that numerous stress-responsive CWPs, particularly chitinase, β-hexosaminida se, glycoside hydrolase, α-galactosidase, pectinesterase, and β-fructofuranosidase, may form strong-interaction networks in maize (**Figure S1**). The stress responsive CWPs may be involved in the cell signaling process under abiotic and biotic stresses.

Moreover, numerous CWPs were not found to be involved in stress responses, such as dirigent protein, expansin, heparanase-like protein, proline rich cell wall protein, and exopolygalacturonase. However, the roles of dirigent protein and expansins in stress responses have been suggested in Arabidopsis (Marowa et al., 2016). The heparanase activity, a process that can lead to invasion by tumor cells (Fux et al., 2009), is strongly implicated in the structural remodeling of the extracellular matrix of animals.

## Cis-Acting Elements in the Genes Encoding Maize CWPs

The hormone abscisic acid (ABA) plays the central role in the adaptation to various stresses (Tuteja, 2007). ABRE element confers the responsiveness to ABA, and the sensitivity to ABA also depends on the presence of myeloblastosis (MYB) and myelocytomatosis (MYC) elements (Kalemba and Pukacka, 2007). Salicylic acid (SA)- and jasmonic acid (JA)-mediated signaling pathways are essential for a plant’s defense response. JA primarily mediates induced systemic resistance to herbivores (Loake and Grant, 2007; Koornneef and Pieterse, 2008), and SA mediates systemic acquired resistance against biotrophic pathogens (Glazebrook, 2005; Thaler et al., 2012).

To find the molecular clues of stress-responsiveness of CWPs, stress- and phytohormone-related cis-acting elements in the promoter regions of their encoding genes were analyzed using Plantcare (http://bioinformatics.psb.ugent.be/webtools/plantcare/html/) and PLACE (http://www.dna.affrc.go.jp/PLACE/signalscan.html). As a result, numerous well-known cis-elements were found in the genes encoding maize CWPs, such as MYB-binding site, ABA responsive element (ABRE), dehydration responsive element (DRE), SA responsive element (TCA), and methyl jasmonate (MeJA) responsive elements (**Table 1**, **Table S3**). This may explain why the stress-responsive CWPs take part in multiple biotic and abiotic stresses.

**Table 1:** Bioinformatic analysis of cis-acting elements in the genes encoding maize cell wall proteins (CWPs).

Cis-acting element	Function	Sequence	Number of CWPs involved^a^
MBS	MYB-binding site	CAACTG or TAACTG	14
DRE	Dehydration responsive element	GCCGAC or ACCGAC	9
LTRE	Low temperature responsive element	CCGAAA	15
WUN	Wound-responsive element	AAATTTCCT	5
TC-rich repeats	Defense and stress responsive element	GTTTTCTTAC or ATTTTCTTCA	6
ABRE	ABA-responsive element	CACGTG or TACGTG	34
TCA-element	Salicylic acid responsive element	CAGAAAAGGA or GAGAAGAATA	14
TGACG	MeJA responsive element	TGACG	33
CGTCA	MeJA responsive element	CGTCA	35

^a^Refer to **Table S3** for the name of CWPs involved.

## Potential Applications of CWPs in Developing Stress-Resistant Crops

As the interface with the environment, CWPs face intense selection pressure to develop new functions or recruit new proteins to the apoplast through gene duplication and retargeting (Rose and Lee, 2010). CWPs present in the cell wall, such as structural proteins, may interact with other wall components *via* noncovalent linkages to form insoluble networks (Spadoni et al., 2006). It is recognized that hydroxyproline-rich glycoproteins (HRGPs) among other CWPs play major roles in a plant’s defense against abiotic and pathogen attacks (Deepak et al., 2010). CWPs present in the extracellular space, particularly between the cell plasma membrane and the cuticle in aerial organs or the suberin layer in roots, may endow the plant surface with waterproof qualities and protection against biotic and abiotic stresses (Thomas et al., 2007; Javelle et al., 2010). The identification and cloning of resistant genes are important prerequisites for the targeted breeding of stress-resistant crops, particularly through gene transfer and genome editing technologies. Almost all known resistant genes encode intracellular proteins (Wu and Wang, 2016), while CWPs have not yet been targeted for the improvement of stress tolerance in crops.

The roles of some CWPs in abiotic stresses have been proved in maize, Arabidopsis, and other plant species. Here, we just referred some examples because we did not aim to comprehensively review earlier studies. In maize root tips, many CWPs, such as α-L-arabinofuranosidase, β-D-glucosidase, β-galactosidase, β-D-xylosidase, and xyloglucan endotransglucosylase/hydrolase (Zhu et al., 2007), in the category of hydrolases respond to water deficit. In Arabidopsis, pectinesterase1 acts as a negative regulator of genes involved in salt stress response (Creighton et al., 2017); pectin methylesterase is required for guarding the cell function in response to heat (Wu et al., 2017); purple acid phosphatase 17 is reducible by ABA, H_2_O_2_, senescence, phosphate starvation, and salt stress (Del Pozo et al., 1999). In rice, β-galactosidase gene responds to ABA and water-stress (Mundy and Chua, 1988) and germin-like proteins are associated with salt stress (Banerjee et al., 2017). In other plants, β-galactosidase is found to be related to heavy metals (Atrooz and Abukhalil, 2016); glycine-rich proteins (Ringli et al., 2001; Mangeon et al., 2010) and cell wall invertase (copper stress, Xu et al., 2017) are stress-induced. Stress upregulates the expression of expansins and xyloglucan-modifying enzymes that can remodel the wall under abiotic stresses (Tenhaken, 2014). In *Medicago truncatula*, xyloglucan endotransglucosylase/hydrolase respond to heavy metal mercury, salinity, and drought stresses (Xuan et al., 2016) through incorporating newly deposited xyloglucan to strengthen cell walls. However, the role of similar maize CWPs under abiotic stresses needs to be characterized.

The role of some CWPs in biotic stresses have been studied in different plant species. Proteomic analysis in Arabidopsis revealed that CWPs such as endochitinase A (ECA), pectinesterase, peroxidase, polygalacturonase, and xyloglucan endotransglucosylase/hydrolase were significantly changed in infected plants by *Pseudomonas syringae*, resulting in enhanced resistance (Jia et al., 2018). Chitinases have antifungal activity against chitin-containing fungal pathogens (Huynh et al., 1992; Volpicella et al., 2017). Overexpressing extensin enhanced the resistance of Arabidopsis toward *Pseudomonas syringae* by promoting cell wall stiffness (Wei and Shirsat, 2006). Particularly, plants respond to pathogen attacks with a wide range of protein inhibitors of cell wall polysaccharide-degrading enzymes (Chivasa et al., 2005; Juge, 2006). In fruits, polygalacturonases and pectatelyases contribute to the softening of fruit. The suppression of these enzymes delays fruit softening and simultaneously confers enhanced resistance to pathogens such as Botrytis (Cantu et al., 2008; Liu et al., 2014). In maize, some CWPs are found to play a role in response of the plant toward biotic stress, e.g., pectinesterase/pectinesterase inhibitor (A0A1D6KNZ1) and xylanase inhibitor protein (Chivasa et al., 2005). Maize aspartyl protease AED3 (B4FMW6) may be involved in the systemic acquired resistance against fungal invasion, and its transcript (*Zm00001d027965*) was increased by a log_2_-fold change of 4.3 in maize infected with *Ustilago maydis*. A few studies have proved that maize ECA has a key role in several biotic stresses due to fungi, bacteria and insect herbivory (Huynh et al., 1992; Moore et al., 2004; Doehlemann et al., 2008; Peethambaran et al., 2010; Mohammadi et al., 2011; Ray et al., 2016), particularly having direct antifungal activity *via* the degradation of fungal cell walls (Wang et al., 2019).

## Conclusion and Future Perspectives

Currently, only limited stress-related genes/proteins are available, and there are no reports on the use of CWPs for enhancing the stress resistance of crops. This study summarizes available knowledge on the proven and predicted functions of maize CWPs by mining information from existing databases. The functions of the highlighted maize CWPs in various stresses, particularly those involved in multiple stresses, should be of special interest in improving stress-resistant maize.

A growing body of evidence support that CWPs are associated with cell wall remodeling during abiotic stresses and pathogen attacks (Houston et al., 2016). Despite their potential roles, the abundance of stress-responsive CWPs may not be sufficient to have substantial effects on crop resistance under severe stresses or multiple stresses. Modifying CWPs with gene transfer and genome editing will be a straightforward approach to develop stress-smart crops, particularly targeting the CWPs common to multiple stresses. The effects of the altered accumulation of these CWPs on plant growth, wall properties, resistance, and other agronomic traits also need to be clarified.

Finally, digging for stress-responsive CWPs will help for developing stress-resistant crops. The in-depth link of proteomics with other omics (particularly metabolomics) and bioinformatics will help the discovery and characterization of stress-tolerant functional genes/CWPs that can be used for the improvement of maize resistance.

## Data Availability Statement

The original contributions presented in the study are included in the article/supplementary material; further inquiries can be directed to the corresponding author.

## Author Contributions

WW conceived the work and edited the manuscript. LN and LL analyzed the data and drafted the manuscript.

## Funding

Our work was supported by the National Natural Science Foundation of China (U1904107).

## Conflict of Interest

The authors declare that the research was conducted in the absence of any commercial or financial relationships that could be construed as a potential conflict of interest.

## Supplementary Material

Supplementary Figure 1 String analysis of possible protein-protein interactions among maize CWPs.
