# Supplementary figures and images for: Digging for Stress-Responsive Cell Wall Proteins for Developing Stress-Resistant Maize
Source: Front Plant Sci. 2020 Sep 25;11:576385. doi: 10.3389/fpls.2020.576385 (PMC7546335; doi:10.3389/fpls.2020.576385)

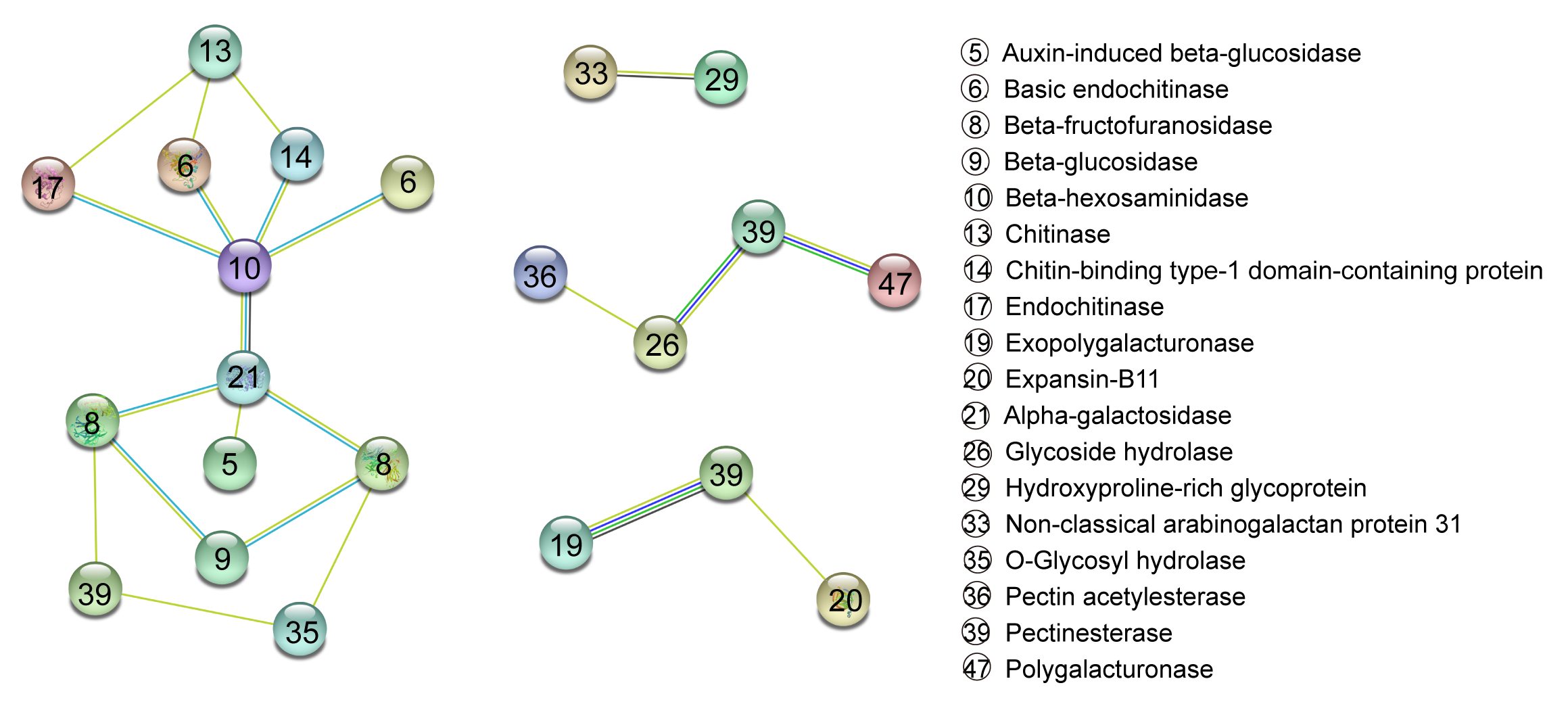

Supplement: Supplementary Figure 1 — String analysis of possible protein-protein interactions among maize CWPs. [file Image_1.tif]
