# Supplementary material for: Digging for Stress-Responsive Cell Wall Proteins for Developing Stress-Resistant Maize
Source: Front Plant Sci. 2020 Sep 25;11:576385. doi: 10.3389/fpls.2020.576385 (PMC7546335; doi:10.3389/fpls.2020.576385)
Supplement: Supplementary file 2 [file DataSheet_1.docx]

**Supplementary dataset 1 |** Maize CWPs Entries Retrieved in UniProtKB

**Note**: Reviewed entries in bold, the number in the parentheses representing sequence number of each protein families; the entries in red having annotation of subcellular locations in UniProtKB.

1. Alpha-L-arabinofuranosidase (64): B4FDR3, B4FXI9, B6T9B9, C0P4P5, K7U6T4, K7UM26, A0A1D6F4L8, A0A1D6F4L9, A0A1D6F4M0, A0A1D6F4M2, A0A1D6F4M3, A0A1D6F4M4, A0A1D6F4M5, A0A1D6F4M7, A0A1D6F4M9, A0A1D6F4N0, A0A1D6F4N1, A0A1D6F4N2, A0A1D6F4N4, A0A1D6F4N6, A0A1D6F4N7, A0A1D6FM22, A0A1D6JP53, A0A1D6JP54, A0A1D6K126, A0A1D6K127, A0A1D6K128, A0A1D6K130, A0A1D6K131, A0A1D6K133, A0A1D6KTM6, A0A1D6KTM7, A0A1D6KTM8, A0A1D6NQE9, A0A1D6NQF0, A0A1D6NQF1, A0A1D6NQF3, A0A1D6NQF5, A0A1D6PRI8, A0A1D6QH20, A0A1D6QH21, A0A1D6QH22, A0A1D6QH23, A0A1D6QH24, A0A1D6QH25, A0A1D6QH26, A0A1D6QH28, A0A1D6QH29, A0A1D6QH30, A0A1D6QH31, A0A1D6QH36, A0A1D6QH38, A0A1R3NDP5, A0A317Y633, A0A3L6DAS4, A0A3L6DDF5, A0A3L6DFF5, A0A3L6DSG2, A0A3L6F147, A0A3L6F279, A0A3L6FJ99, A0A3L6FUF4, A0A3L6FYQ6, A0A3L6GC12
2. Alpha-L-fucosidase 2 (6): A0A1D6KCJ1, A0A1D6KCJ0, A0A1D6KCI9, A0A1D6KCI8, A0A1D6KCI7, A0A317Y497
3. Ankyrin repeat family protein (4): A0A1D6GGR9, B6TPI5, C0P943, C0P9I4
4. Aspartyl protease/Aspartic proteinase nepenthesin (7): B4FMW6, B4G037, B4G1Q7, B6SJT4, B6TE66, C0P9F8, C0PB10
5. Auxin-induced β-glucosidase (1): B6SWK9
6. Basic endochitinase (12): B6SZC6, B6T6W1, B6TFQ3, B6TR38, C0P3M6, K7VXP1, A0A1D6GWN1, A0A1D6GWN3, A0A1D6K­7T5, A0A1D6LKX7, A0A1D6LMS1, A0A1D6PVW4
7. Beta-D-xylosidase (5): B4F8R5, B8A1R0, A0A1D6E4T8, A0A1D6EGK8, A0A1D6JFG2
8. Beta-fructofuranosidase, cell wall isozyme (3): **P49174**, A0A1D6H9Z1, A0A3L6EFD5
9. Beta-glucosidase (1): A0A1D6GTQ2
10. Beta-hexosaminidase (2): B6ST04, A0A1D6LHI9
11. Carbohydrate-binding-like fold (1): A0A1D6FHT0
12. Cell wall invertase (7): Q9SBI2, Q9SPK0, Q9ZTL2, Q9ZTQ4, Q9ZTQ5, A0A1D6DUP5, A0A1D6J6P6
13. Chitinase (3): D0EM57, A0A317Y748, A0A317Y7C2
14. Chitin-binding type-1 domain-containing protein (2): B4FK87, A0A1X7YIJ7
15. Dirigent protein (1): B6TQI8
16. DUF1005 family protein (1): B6SZT8
17. Endochitinase (7): B4FTS6, B6SZA3, B6TEL0, B6TT00, C0PKN5, **P29022**, A0A1D6E7B3
18. Eukaryotic aspartyl protease family protein (1): A0A1D6DSN9
19. Exopolygalacturonase (18): B4FUB7, B6T4T5, B6TAW9, C4JBB6, **P26216**, **P35338**, **P35339**, A0A317Y5Q5, A0A3L6DI3, A0A3L6E932, A0A3L6E950, A0A3L6EDM2, A0A3L6EHF9, A0A3L6ENL7, A0A3L6EUF7, A0A3L6F4K1, A0A3L6F7R4, A0A3L6FM61
20. Expansin (109): B4F8B6, B4FEH6, B4FLU4, B4FPA9, B4FRP2, B4FWR8, B4FYF7, B6SGJ2, B6T5Z7, B6T7X4, B6TJY0, B6TP92, B6TQR2, B6UA99, B8A1G1, C0HEC1, C0HEY5, C0P2N8, C0P890, C0P9D0, C0PJW8, C4JBZ0, K7VDB7, K7VT40, K7WH77, **P0C1Y5**, **P58738**, **Q07154**, **Q1ZYQ8**, Q94KT3, Q94KT5, Q94KT6, Q94KT7, A0A096TYJ9, A0A1D6H550, A0A1D6H557, A0A1D6H579, A0A1D6H581, A0A1D6HK98, A0A1D6JBP1, A0A1D6JNZ8, A0A1D6JNZ9, A0A1D6JP00, A0A1D6KM59, A0A1D6KM60, A0A1D6KUN7, A0A1D6KUP3, A0A1D6KUP4, A0A1D6KUP5, A0A1D6KUP6, A0A1D6KUP7, A0A1D6LEA6, A0A1D6LEH7, A0A1D6LEH8, A0A1D6LEH9, A0A1D6LEI0, A0A1D6LEI2, A0A1D6LEI3, A0A1D6LEI4, A0A1D6LEI5, A0A1D6LLK0, A0A1D6M6L1, A0A1D6MPF4, A0A1D6MPF6, A0A1D6MQG9, A0A1D6QEM7, A0A317Y0P1, A0A317Y0P9, A0A317Y1K1, A0A317Y1X3, A0A317Y204, A0A317Y390, A0A317Y3L4, A0A317Y881, A0A317YB29, A0A317YBV3, A0A317YDN7, A0A317YHC7, A0A317YJE0, A0A3L6D8P5, A0A3L6DA75, A0A3L6DEK2, A0A3L6DF32, A0A3L6DGY1, A0A3L6E6H6, A0A3L6E7R1, A0A3L6E7W5, A0A3L6E882, A0A3L6EER6, A0A3L6EGY3, A0A3L6EHG3, A0A3L6EHM1, A0A3L6EKA7, A0A3L6EMW2, A0A3L6ESB1, A0A3L6EVR9, A0A3L6EW90, A0A3L6EWK9, A0A3L6EY84, A0A3L6F4H0, A0A3L6F5I5, A0A3L6FB36, A0A3L6FCH0, A0A3L6FFL8, A0A3L6FL84, A0A3L6FUS7, A0A3L6G407, A0A3L6G9X2, A0A3L6GC21
21. Galactosidase, *α-type* (11): B4FA27, C0PAL4, A0A1D6EFC8, A0A1D6F7Z2, A0A1D6HYF2, A0A1D6JPI1, A0A1D6JWU1, A0A1D6KS86, A0A1D6KS92, A0A1D6PKF8, A0A1D6QBS8
22. Galactosidase, *β-type* (16): B4F9J1, B8A2F0, C0P3T5, K7V4R8, A0A1D6QQE9, A0A1D6EU33, A0A1D6FPT2, A0A1D6IPT8, A0A1D6JPS0, A0A1D6MYX1, A0A1D6N5T1, A0A1D6N5U0, A0A1D6NGI9, A0A1D6NKE5, A0A1D6PQ20, A0A1D6JWI7
23. Germin-like protein (subfamily 1, 2, 3 and T) (42): B4FAV5, B4FRS8, B4FUT3, B4FY73, B6UEL1, K7TT00, K7TT05, K7TT15, K7TWM7, K7TWN5, K7U0Q4, K7U0Q9, K7UAX3, K7UAX9, K7UAY3, K7UAZ2, K7UDN3, K7URE9, K7URF1, K7URF5, K7URF9, K7W1E7, Q6TM44, A0A1D6DPN9, A0A1D6DZA4, A0A1D6ELU8, A0A1D6H1F2, A0A1D6H9X6, A0A1D6HTL7, A0A1D6J4J1, A0A1D6JQE7, A0A1D6KN97, A0A1D6KN98, A0A1D6L886, A0A1D6M3P4, A0A1D6N137, A0A1D6NEI1, A0A1D6PUN9, A0A1D6PUP6, A0A1D6PUQ1, A0A1D6Q1Q1, A0A1D6QI94
24. Glycine-rich cell wall structural protein (14): B4FXY6, B6SPN2, B6SR24, B6ST85, B6TEE0, B6TFA1, B6TY69, B6TZT1, B6U1E3, B6U4A6, B6U6I9, B6UGA1, A0A1D6HBT3, A0A1D6KU97
25. Glyco_hydro_19_cat domain-containing protein (5): C0HEI0, B6SZN3, C0P306, B8A247, A0A1D6JS63
26. Glycoside hydrolase (family 28) (2): B6TX01, B6TXJ8
27. Group 3 pollen allergen (2): K7U2A7, Q7XBA3
28. Heparanase-like protein 1 (8): B4FGA1, B6SRR5, B6U6D3, A0A1D6HNK6, A0A1D6KEH1, A0A1D6NEB8, A0A1D6NTX9, A0A1D6QBZ8
29. Hydroxyproline-rich glycoprotein (HRGP) (2): B4FHE8, Q42366
30. Leucine-rich repeat (LRR) family protein (1): K7U7Y3
31. Malate dehydrogenase (1): B4FRJ1
32. NADH-cytochrome b5 reductase (1): B6TCK3
33. Non-classical arabinogalactan protein 31/ Pistil-specific extensin-like (2): A0A3L6FGF2/B6UHE3
34. Nudix hydrolase domain-containing protein (1): A0A1D6LN55
35. O-Glycosyl hydrolase (12): B4FL15, B4FQC1, B4FU19, B6STC4, C0P2R5, C0PI20, K7V329, K7WB31, A0A1D6LN55, A0A1D6LPQ2, A0A1D6PTZ2, A0A1D6QK01
36. Pectin acetylesterase (66): B4F9N3, B4F9X6, B4FL51, B4FVG6, B4FZC4, B6T178, B6TXR7, B6U063, B6U7Q4, B8A2J2, C0HHW3, C0P5Y1, C0PGF7, C0PGW1, C0PML3, C4J1M5, K7UYB4, K7V2C5, K7VC42, K7VY73, A0A1D6E0M4, A0A1D6E0M5, A0A1D6E0M6, A0A1D6E0M8, A0A1D6FVP1, A0A1D6FVP2, A0A1D6G797, A0A1D6G798, A0A1D6G799, A0A1D6G7A0, A0A1D6HH87, A0A1D6HH88, A0A1D6IKN4, A0A1D6IKN6, A0A1D6IKN7, A0A1D6IKN8, A0A1D6IKN9, A0A1D6IXV8, A0A1D6K689, A0A1D6LVW0, A0A1D6MMW1, A0A1D6MMW2, A0A1D6MS67, A0A1D6MS68, A0A1D6MZZ9, A0A1D6N558, A0A1D6NME3, A0A1D6NMF0, A0A1D6NMF3, A0A1D6P731, A0A1D6P732, A0A317Y4U1, A0A3L6DJ08, A0A3L6DN84, A0A3L6DQP4, A0A3L6E4G9, A0A3L6E5A0, A0A3L6EJM5, A0A3L6EK46, A0A3L6ETB2, A0A3L6F5A7, A0A3L6FC08, A0A3L6FKB6, A0A3L6FKX2, A0A3L6FTD1, A0A3L6G6D8
37. Pectin lyase (51): B4F828, B4FQ16, B4FX45, B4G0U5, C0P541, C0PDS0, K7VYM8, A0A1D6FHK6, A0A1D6FIZ2, A0A1D6FL95, A0A1D6FZH5, A0A1D6FZH6, A0A1D6H437, A0A1D6H438, A0A1D6H446, A0A1D6H451, A0A1D6H453, A0A1D6H471, A0A1D6HWL6, A0A1D6I580, A0A1D6JM54, A0A1D6KD22, A0A1D6LAC6, A0A1D6LAC7, A0A1D6LAD1, A0A1D6LRB8, A0A1D6LRC6, A0A1D6LRD7, A0A1D6LRF1, A0A1D6LRF3, A0A1D6LRF7, A0A1D6LRF9, A0A1D6LRK1, A0A1D6LRM6, A0A1D6LRN2, A0A1D6LRN5, A0A1D6LRN7, A0A1D6LRN9, A0A1D6LRP3, A0A1D6LRP4, A0A1D6LRP6, A0A1D6LRQ5, A0A1D6LRQ6, A0A1D6LRQ7, A0A1D6LRQ8, A0A1D6LTC8, A0A1D6MK53, A0A1D6PNR8, A0A1D6Q047, A0A1D6QP73, A0A1Q1AZD7
38. Pectin methylesterase (5): K7W5K6, A0A1D6J7X2, A0A1D6J7X3, A0A1D6J7X8, A0A1D6J7Y2
39. Pectinesterase (108): B4F9U3, B4FCJ7, B4FCM1, B4FCY2, B4FI46, B4FKQ3, B4FKZ4, B4FRR6, B6SZL3, B6TMD5, B6TZ43, B6TZE6, B6U6B3, B8A0X6, B8A1C3, B8A2X5, C0HGE0, C0PFP7, C0PMP7, C4IZT1, C4J3B1, C4J543, K7TP23, K7UUE2, O24596, A0A1D6E3U7, A0A1D6E3U8, A0A1D6E3U9, A0A1D6E3V3, A0A1D6E3V6, A0A1D6E967, A0A1D6E971, A0A1D6E973, A0A1D6E975, A0A1D6E976, A0A1D6E979, A0A1D6E980, A0A1D6E981, A0A1D6E982, A0A1D6E985, A0A1D6F4U1, A0A1D6F4U3, A0A1D6FHT9, A0A1D6FU21, A0A1D6FU22, A0A1D6G099, A0A1D6GCB2, A0A1D6GCB3, A0A1D6GCB4, A0A1D6H4M1, A0A1D6HB34, A0A1D6I082, A0A1D6I4Y9, A0A1D6IJ74, A0A1D6IN03, A0A1D6IP48, A0A1D6IYN8, A0A1D6JFI4, A0A1D6JFI5, A0A1D6JFI7, A0A1D6JFI9, A0A1D6JFJ0, A0A1D6JFJ1, A0A1D6JFJ5, A0A1D6JFJ7, A0A1D6JVC6, A0A1D6JVC7, A0A1D6JVC8, A0A1D6JZZ9, A0A1D6KDB9, A0A1D6KE17, A0A1D6KNK0, A0A1D6MAL2, A0A1D6MAL3, A0A1D6MAL4, A0A1D6MAL5, A0A1D6MAL6, A0A1D6MRY9, A0A1D6N7W7, A0A1D6NCW4, A0A1D6NCW6, A0A1D6NP08, A0A1D6PJ96, A0A1D6PN89, A0A1D6PN90, A0A1D6PN91, A0A1D6PN92, A0A1D6PN93, A0A1D6PN94, A0A1D6PN95, A0A1D6PZP0, A0A1Q1BFQ0, A0A317Y1Q1, A0A317Y8F9, A0A317Y8P7, A0A317YA46, A0A317YGS5, A0A317YIS6, A0A3L6DIV2, A0A3L6DMF5, A0A3L6DST7, A0A3L6DSY6, A0A3L6DY29, A0A3L6E441, A0A3L6F9R9, A0A3L6FDM2, A0A3L6FUS0, A0A3L6GCM3
40. Pectinesterase/pectinesterase inhibitor (4): A0A1D6KNZ1, A0A1D6N5J3, A0A1D6P828, A0A3L6DFM6
41. Pepsin A (3): B6SJD9, C0PN95, A0A1D6E4P0
42. Peptidase A1 domain-containing protein (1): B4G1Q7
43. Peroxidase (82): **A5H8G4**, B4F7T9, B4FBH0, B4FG25, B4FH35, B4FH68, B4FHG3, B4FK72, B4FKG0, B4FNL8, B4FRD6, B4FSW5, B4FU88, B4FVT1, B4FY83, B4FYH1, B4G1C4, B6SIA9, B6SIU4, B6TWB1, C0HFN4, C0HHA6, C0HIT1, C0P4S1, C0PKS1, C4IZA5, D7NLB3, K7TID5, K7TMB0, K7TMC0, K7U151, K7U159, K7UF86, K7UG68, K7UQ82, K7USX8, K7V8K5, K7VDC0, K7VGN1, K7VGN6, K7VQB0, **Q9FEQ8**, A0A1D6E530, A0A1D6E534, A0A1D6F256, A0A1D6F265, A0A1D6FHV4, A0A1D6FP28, A0A1D6FQT9, A0A1D6FUY7, A0A1D6FUY8, A0A1D6H7N5, A0A1D6HQQ8, A0A1D6HX87, A0A1D6I695, A0A1D6IBW1, A0A1D6IKV7, A0A1D6IKV8, A0A1D6IKV9, A0A1D6IKW0, A0A1D6IKW1, A0A1D6IKW2, A0A1D6IKX3, A0A1D6J1L2, A0A1D6JF04, A0A1D6JNY2, A0A1D6K431, A0A1D6K433, A0A1D6K434, A0A1D6K8V8, A0A1D6KQI0, A0A1D6LE55, A0A1D6LFH4, A0A1D6LYW3, A0A1D6MRI4, A0A1D6MRI5, A0A1D6MSC0, A0A1D6MYJ1, A0A1D6N0K1, A0A1D6N0K3, A0A1D6N9N4, A0A1D6NZH6
44. Peroxiredoxin (1): B6T2Y1
45. Plant L-ascorbate oxidase (1): A0A1D6P233
46. Polyamine oxidase 1 (1): **O64411**
47. Polygalacturonase (69): B4F8V7, B4FAG1, B4FFH7, B4FMP4, B4FWD4, B4FY93, B4FZT4, B4G1N7, B6T6X1, B6TDS0, B6TMM0, B6TPQ7, B6TUZ2, B6TYD6, B6U0W6, B6U5Y4, B6UB39, B8A0S9, B8A0S9, B8A1W7, C0P4S3, C0P8E0, C0PIY8, C0PKK6, C4J3A3, C4J705, C4JBH3, K7VC52, K7VKI0, K7W8T8, A0A1D6FI10, A0A1D6FL13, A0A1D6G0A5, A0A1D6L8J1, A0A1D6L8J2, A0A1D6L8S3, A0A1D6MBI7, A0A1D6MBI8, A0A1D6MBI9, A0A1D6MSF0, A0A1D6NHT4, A0A1D6QRB3, A0A1D6QRB5, A0A317Y923, A0A317Y983, A0A317YGJ9, A0A317YH27, A0A3L6D5R1, A0A3L6DD25, A0A3L6DH97. A0A3L6DPM5, A0A3L6DQZ3, A0A3L6DSL2, A0A3L6DV79, A0A3L6DX62, A0A3L6DX72, A0A3L6DXF5, A0A3L6ECQ5, A0A3L6EGR8, A0A3L6ER92, A0A3L6ESU7, A0A3L6EVR8, A0A3L6EYK9, A0A3L6F8E8, A0A3L6FE81, A0A3L6FG77, A0A3L6FGT4, A0A3L6FHN6, A0A3L6FMD2
48. Proline and lysine rich protein (4): K9L7F0, K9L7S1, K9L8D5, Q9ZNY1
49. Protein EXORDIUM-like 3 (1): K7V7C0
50. Purple acid phosphatase (2): B4FR72, B6SWS9
51. Pyrroline-5-carboxylate reductase (1): Q4TZJ2
52. Subtilisin-like protease SBT2.6 (1): C0P6H8
53. UDP-arabinopyranose mutase (2): B4FX25, **P80607**
54. Uncharacterized protein (1): B4FUQ3
55. Vegetative cell wall protein (4): B6SY74, B6T5K5, B6U8J0, A0A1D6MR59
56. Xyloglucan endotransglucosylase/hydrolase (80): B4F837, B4F9C6, B4FAV6, B4FBM2, B4FE99, B4FHS5, B4FSS4, B4FTH5, B4FWJ4, B4FWW4, B4G1Z2, B6T2W7, B6T9E1, B6T9H5, B6TDC2, B6TEW5, B6TH17, B6TJ72, B6TK97, B6TM34, B6TQ18, B6TR01, B6TWP1, B6TX02, B6U5W7, B8A0K3, C0HJ15, C0P2K7, C0P6G9, C0P7W9, C0PC72, C0PCM5, C0PL00, E1U818, K7TQ85, K7TW38, K7U6H7, K7U9U0, K7USG7, K7USS3, K7V8Z6, K7VQE7, K7W8V9, Q42446, Q5JZX2, A0A096T7G6, A0A1D6DXK8, A0A1D6E0F4, A0A1D6E0F6, A0A1D6E0F8, A0A1D6GUH2, A0A1D6GUH3, A0A1D6GUL2, A0A1D6H4U7, A0A1D6IZ08, A0A1D6K9K3, A0A1D6PF72, A0A1D6Q7L1, A0A1D6QTW8, A0A1D6QTX0, A0A317Y4A5, A0A317YG22, A0A317YGM9, A0A3L6DB05, A0A3L6DEA6, A0A3L6DFU1, A0A3L6E5M8, A0A3L6EJS3, A0A3L6EZA2, A0A3L6F3T2, A0A3L6F6F2, A0A3L6F7U3, A0A3L6FNT1, A0A3L6FVI4, A0A3L6FWX7, A0A3L6G4F1, A0A3L6G4Z0, A0A3L6GDI3, A0A1D6IYZ9, A0A1D6IZ02
