# Supplementary material for: Digging for Stress-Responsive Cell Wall Proteins for Developing Stress-Resistant Maize
Source: Front Plant Sci. 2020 Sep 25;11:576385. doi: 10.3389/fpls.2020.576385 (PMC7546335; doi:10.3389/fpls.2020.576385)
Supplement: Supplementary file 3 [file Table_1.docx]

**Table S1 |** Comparison of protein compositions of CWPs between maize and rice

| **Functional catalog** | **Maize (number)** | **Rice (number)** |
| --- | --- | --- |
| Proteins acting on cell wall carbohydrate metabolism | Alpha-l-arabinofuranosidase (64)  Auxin-induced beta-glucosidase (1)  Beta-D-xylosidase (5)  Beta-fructofuranosidase (3)  Beta-glucosidase (1)  Beta-hexosaminidase (2)  Cell wall invertase (7)  Glycoside hydrolase (2)  O-Glycosyl hydrolase (5)  Pectin acetylesterase (66)  UDP-arabinopyranose mutase (1)  Xyloglucan endotransglucosylase-hydrolase (84) | Glycoside hydrolase (70)  Expansin (8)  Carbohydrate esterase (3)  Polysaccharide lyase (1) Lectin (1)  LRR protein (7)  Pectin methyl esterase inhibitor (2)  Protease inhibitor (5)  GDSL family (7)  Lipid transfer protein (12)  COBRA-like (1)  Fasciclin-like arabinogalactan protein (4) |
| Oxido-reductases | Peroxidase (83)  NADH-cytochrome b5 reductase (1)  Peroxiredoxin (1)  L-ascorbate oxidase (1)  Polyamine oxidase (1)  Pyrroline-5-carboxylate reductase (1) | Class III peroxidase (26)  Blue copper binding protein (5)  Multicopper oxidase (3)  Berberine-bridge oxido-reductase-like (2) |
| Protein metabolic process | Glycine-rich cell wall structural protein (14)  Ankyrin repeat family protein (4)  Aspartyl protease (7)  Eukaryotic aspartyl protease (1)  Peptidase A1 domain-containing protein (1)  Subtilisin-like protease (2) | Aspartyl protease (14)  Cys protease (4)  Ser carboxypeptidase (7)  Ser protease (6)  Metallopeptidase (1)  LRR-RLK (1) |
| Chitin catabolic process | Basic endochitinase (12)  Chitinase (3)  Endochitinase (7)  Pectinesterase (94) |  |
| Structural proteins | Pectinesterase-pectinesterase inhibitor (4) | Glycine-rich protein (2)  Leucine-rich repeat extensin (5)  Threonine-hydroxyproline-rich glycoprotein (2) |
| Miscellaneous proteins | Germin-like protein (42)  Purple acid phosphatase (2) | Thaumatin (6)  Metallophosphoesterase (1)  Germin (5)  Gibberellic acid-stimulated Arabidopsis (GASA) protein (3)  Purple acid phosphatase (1) |
