# Supplementary material for: Digging for Stress-Responsive Cell Wall Proteins for Developing Stress-Resistant Maize
Source: Front Plant Sci. 2020 Sep 25;11:576385. doi: 10.3389/fpls.2020.576385 (PMC7546335; doi:10.3389/fpls.2020.576385)
Supplement: Supplementary file 4 [file Table_2.docx]

Table S2 | GO annotation of representative maize CWPs and stress-inducible gene expressions

| **Protein name** | **UniProt ID** | **Gene ID** | **Response to biotic stress** | | | **Response to abiotic stress** | | | | | |
| --- | --- | --- | --- | --- | --- | --- | --- | --- | --- | --- | --- |
|  |  |  | Fungal invasion | Insect attack | Bacterium invasion | Acidic soil | Drought | Cold | Heat | Waterlogging | High nitrate stimulation |
| Alpha-L-arabinofuranosidase 1 | A0A1D6K126 | Zm00001d028952 | √ | √ |  | √ |  |  |  |  |  |
| Ankyrin repeat family protein | A0A1D6GGR9 | zm00001d013242 |  |  |  |  | √ | √ |  |  |  |
| Aspartyl protease AED3 | B4FMW6 | zm00001d027965 | √ |  |  | √ | √ | √ | √ | √ |  |
| Auxin-induced β-glucosidase | B6SWK9 | Zm00001d048669 | √ | √ |  |  |  | √ |  | √ | √ |
| Beta-D-xylosidase | B4F8R5 | zm00001d018080 | √ |  |  |  | √ |  | √ | √ |  |
| Beta-fructofuranosidase | P49174 | zm00001d016708 | √ | √ |  | √ | √ | √ |  | √ |  |
| Beta-glucosidase | A0A1D6GTQ2 | zm00001d014489 | √ |  |  |  |  | √ | √ |  |  |
| Beta-hexosaminidase | B6ST04 | zm00001d035598 | √ |  |  | √ |  |  |  |  |  |
| Cell wall invertase | Q9ZTQ5 | zm00001d025355 | √ |  |  |  | √ |  |  |  |  |
| Chitinase | D0EM57 | zm00001d003190 | √ | √ |  | √ | √ |  | √ | √ |  |
| Eukaryotic aspartyl protease | A0A1D6DSN9 | Zm00001d001771 | √ |  |  | √ | √ |  |  | √ |  |
| Germin-like protein | B4FAV5 | zm00001d008210 | √ |  |  | √ | √ |  | √ | √ |  |
| Glycine-rich cell wall structural protein | B4FXY6 | zm00001d017032 | √ |  |  |  | √ | √ |  | √ |  |
| Glycoside hydrolase | B6TX01 | zm00001d027441 | √ |  |  |  | √ |  |  |  |  |
| NADH-cytochrome b5 reductase | B6TCK3 | zm00001d039656 | √ | √ |  |  |  |  |  | √ |  |
| O-Glycosyl hydrolase superfamily protein | K7V329 | zm00001d052792 | √ |  |  |  | √ | √ | √ | √ |  |
| Pectin acetylesterase | B4F9X6 | zm00001d022258 | √ | √ |  |  | √ |  |  | √ |  |
| Pectin lyase-like superfamily protein | B4F828 | zm00001d009341 | √ |  |  |  |  |  |  |  |  |
| Peptidase A1 domain-containing protein | B4G1Q7 | zm00001d001771 | √ |  |  | √ | √ |  | √ | √ |  |
| Peroxidase | A5H8G4 | zm00001d040702 | √ |  |  |  | √ | √ | √ | √ |  |
| Peroxiredoxin | B6T2Y1 | zm00001d046682 | √ | √ | √ |  |  |  |  | √ |  |
| Plant L-ascorbate oxidase | A0A1D6P233 | zm00001d046330 | √ |  |  |  |  | √ | √ | √ |  |
| Purple acid phosphatase | B4FR72 | zm00001d046593 | √ |  |  |  | √ |  |  | √ |  |
| Subtilisin-like protease SBT26 | C0P6H8 | zm00001d036483 | √ |  |  |  | √ |  | √ | √ |  |
| UDP-arabinopyranose mutase | P80607 | zm00001d013751 | √ | √ |  | √ | √ | √ | √ | √ |  |
| Xyloglucan endotransglucosylase/hydrolase | B6T2W7 | zm00001d029814 |  |  |  |  |  | √ | √ | √ |  |
