# Supplementary material for: Digging for Stress-Responsive Cell Wall Proteins for Developing Stress-Resistant Maize
Source: Front Plant Sci. 2020 Sep 25;11:576385. doi: 10.3389/fpls.2020.576385 (PMC7546335; doi:10.3389/fpls.2020.576385)
Supplement: Supplementary file 5 [file Table_3.docx]

**Table S3** **|** Bioinformatic analysis of cis-acting elements in the genes encoding maize CWPs

| **Protein name** | **MBS** | **DRE** | **LTRE** | **WUN-motif** | **TC-rich repeats** | **ABRE** | **TCA-element** | **TGACG-motif** | **CGTCA-motif** |
| --- | --- | --- | --- | --- | --- | --- | --- | --- | --- |
| *Expansin* |  |  |  |  |  | √ |  | √ | √ |
| *Pectinesterase* |  |  | √ |  |  |  |  |  |  |
| *Peroxidase* |  |  |  |  |  | √ |  | √ | √ |
| *Xyloglucan endotransglucosylase/hydrolase* | √ |  | √ |  |  | √ | √ | √ | √ |
| *Polygalacturonase* |  |  |  |  |  | √ | √ | √ | √ |
| *Pectin acetylesterase* |  | √ | √ |  |  | √ |  | √ | √ |
| *Alpha-L-arabinofuranosidase* |  |  |  | √ |  |  | √ |  |  |
| *Pectin lyase* | √ |  |  | √ |  |  | √ |  |  |
| *Germin-like protein* | √ |  |  |  |  | √ |  | √ | √ |
| *Exopolygalacturonase* |  |  |  |  |  |  |  | √ | √ |
| *Galactosidase, β-type* |  |  | √ |  |  | √ |  |  |  |
| *Glycine-rich cell wall structural protein* | √ | √ |  |  | √ | √ | √ |  |  |
| *Basic endochitinase* |  |  |  | √ |  |  |  | √ | √ |
| *O-Glycosyl hydrolase* | √ |  |  |  |  | √ |  | √ | √ |
| *Galactosidase, α-type* |  |  | √ |  |  | √ |  | √ | √ |
| *Heparanase-like protein 1* | √ |  |  |  |  | √ |  | √ | √ |
| *Aspartyl protease/Aspartic proteinase nepenthesin* | √ |  | √ |  |  | √ |  |  | √ |
| *Cell wall invertase* |  | √ |  |  | √ | √ |  | √ | √ |
| *Endochitinase* |  |  |  |  |  | √ | √ | √ | √ |
| *Alpha-L-fucosidase 2* | √ |  |  |  |  | √ |  |  |  |
| *Beta-D-xylosidase* | √ |  |  |  |  | √ |  | √ | √ |
| *Pectin methylesterase* |  |  |  |  |  | √ |  | √ | √ |
| *Ankyrin repeat family protein* | √ | √ |  | √ |  |  | √ | √ | √ |
| *Proline and lysine rich protein* |  | √ |  |  |  | √ |  | √ | √ |
| *Vegetative cell wall protein* |  |  |  |  | √ |  |  | √ | √ |
| *Beta-fructofuranosidase* | √ |  |  |  |  | √ |  |  |  |
| *Pepsin A* |  | √ |  |  | √ |  | √ | √ | √ |
| *Beta-hexosaminidase* |  |  |  |  |  | √ | √ | √ | √ |
| *Glycoside hydrolase* |  |  |  |  |  | √ |  | √ | √ |
| *Group 3 pollen allergen* |  |  | √ |  |  |  |  |  |  |
| *Hydroxyproline-rich glycoprotein* |  |  | √ |  |  | √ |  | √ | √ |
| *Purple acid phosphatase* |  |  | √ |  |  |  |  | √ | √ |
| *UDP-arabinopyranose mutase* |  |  | √ |  |  | √ | √ |  |  |
| *Auxin-induced β-glucosidase* |  |  |  |  |  | √ | √ |  | √ |
| *Beta-glucosidase* |  |  |  |  |  |  | √ | √ | √ |
| *Carbohydrate-binding-like fold* |  |  |  |  |  | √ |  | √ | √ |
| *Dirigent protein* | √ | √ | √ |  |  | √ |  | √ | √ |
| *DUF1005 family protein* | √ |  | √ |  |  |  | √ | √ | √ |
| *Eukaryotic aspartyl protease family protein* |  | √ |  |  |  | √ |  |  |  |
| *Leucine-rich repeat (LRR) family protein* |  | √ | √ |  | √ | √ |  | √ | √ |
| *Malate dehydrogenase* |  |  |  |  |  | √ |  | √ | √ |
| *NADH-cytochrome b5 reductase* | √ |  | √ | √ |  | √ |  | √ | √ |
| *Nudix hydrolase domain-containing protein* |  |  | √ |  |  |  |  |  |  |
| *Plant L-ascorbate oxidase* |  |  |  |  |  | √ | √ | √ | √ |
| *Protein EXORDIUM-like 3* |  |  |  |  |  | √ |  | √ | √ |
| *Pyrroline-5-carboxylate reductase* |  |  |  |  |  | √ |  |  |  |
| *Subtilisin-like protease SBT2.6* |  |  |  |  | √ | √ |  | √ | √ |

Note: The symbol √ indicates the presence of a specific sequence in the promoter region of the encoding gene of a protein.
